# Bis[tetra­kis­(pyridin-2-yl)methane-κ^3^ *N*,*N*′,*N*′′]cobalt(II) tetra­kis­(thio­cyanato-κ*N*)cobaltate(II) methanol monosolvate

**DOI:** 10.1107/S160053681400289X

**Published:** 2014-02-15

**Authors:** Yuya Tsunezumi, Kouzou Matsumoto, Shinya Hayami, Akira Fuyuhiro, Satoshi Kawata

**Affiliations:** a Department of Chemistry, Faculty of Science, Fukuoka University, Nanakuma, Jonan-ku, Fukuoka 814-0180, Japan; b Institute of Natural Sciences, Senshu University, Higashimita, Tama-ku, Kanagawa 214-8580, Japan; c Department of Chemistry, Graduate School of Science and Technology, Kumamoto University, Kurokami, Kumamoto 860-8555, Japan; d Department of Chemistry, Graduate School of Science, Osaka University, Toyonaka, Osaka 560-0043, Japan

## Abstract

The title complex, [Co(C_21_H_16_N_4_)_2_][Co(NCS)_4_]·CH_3_OH, consists of one [Co{C(py)_4_}_2_]^2+^ complex cation [C(py)_4_ = tetra­kis­(pyridin-2-yl)methane], one [Co(NCS)_4_]^2−^ complex anion and a methanol solvent mol­ecule. In the cation, the Co^II^ atom is coordinated by six N atoms of two C(py)_4_ ligands in a distorted octa­hedral geometry. In the anion, the Co^II^ atom is coordinated by the N atoms of four NCS^−^ ligands in a distorted tetra­hedral geometry. The methanol mol­ecule is disordered and was modelled over three orientations (occupancies 0.8:0.1:0.1). There are two weak hydrogen-bond-like inter­actions between the methanol solvent mol­ecule and NCS^−^ ligands of the anion [O⋯S = 3.283 (3) and 3.170 (2) Å].

## Related literature   

For details of polypyridyl complexes, see: Hayami *et al.* (2011 ▶); Kalyanasundaram & Grätzel (1998 ▶). For Co–tris­(pyridin-2-yl)methane complexes, see: Adam *et al.* (1997 ▶). For tetra­kis­(pyridin-2-yl)methane, see: Matsumoto *et al.* (2003 ▶).
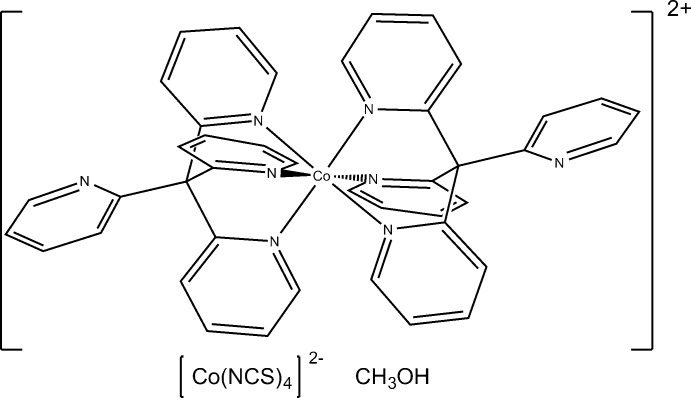


## Experimental   

### 

#### Crystal data  

[Co(C_21_H_16_N_4_)_2_][Co(NCS)_4_]·CH_4_O
*M* *_r_* = 1026.96
Monoclinic, 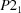

*a* = 8.903 (3) Å
*b* = 21.732 (6) Å
*c* = 12.335 (4) Å
β = 108.480 (4)°
*V* = 2263.4 (11) Å^3^
*Z* = 2
Mo *K*α radiation
μ = 0.97 mm^−1^
*T* = 100 K
0.70 × 0.20 × 0.10 mm

#### Data collection  

Rigaku Saturn724 diffractometer
Absorption correction: multi-scan (*REQAB*; Rigaku, 1998 ▶) *T*_min_ = 0.796, *T*_max_ = 0.908
35095 measured reflections
10355 independent reflections
9357 reflections with *F* ^2^ > 2.0σ(*F* ^2^)
*R*_int_ = 0.039

#### Refinement  

*R*[*F* ^2^ > 2σ(*F* ^2^)] = 0.034
*wR*(*F* ^2^) = 0.071
*S* = 1.01
10355 reflections
596 parameters
4 restraints
H-atom parameters constrained
Δρ_max_ = 0.49 e Å^−3^
Δρ_min_ = −0.35 e Å^−3^
Absolute structure: Flack (1983 ▶), 5024 Friedel pairs
Absolute structure parameter: −0.008 (8)

### 

Data collection: *CrystalClear* (Rigaku, 2008 ▶); cell refinement: *CrystalClear*; data reduction: *CrystalClear*; program(s) used to solve structure: *SIR92* (Altomare *et al.*, 1994 ▶); program(s) used to refine structure: *SHELXL97* (Sheldrick, 2008 ▶); molecular graphics: *CrystalStructure* (Rigaku, 2010 ▶); software used to prepare material for publication: *CrystalStructure*.

## Supplementary Material

Crystal structure: contains datablock(s) General, I. DOI: 10.1107/S160053681400289X/pk2517sup1.cif

Structure factors: contains datablock(s) I. DOI: 10.1107/S160053681400289X/pk2517Isup2.hkl

CCDC reference:

Additional supporting information:  crystallographic information; 3D view; checkCIF report

## Figures and Tables

**Table 1:** Selected bond lengths (Å)

Co1—N1	2.169 (3)
Co1—N2	1.999 (2)
Co1—N3	1.923 (3)
Co1—N5	2.166 (2)
Co1—N6	1.996 (2)
Co1—N7	1.923 (3)

**Fig. 1.**
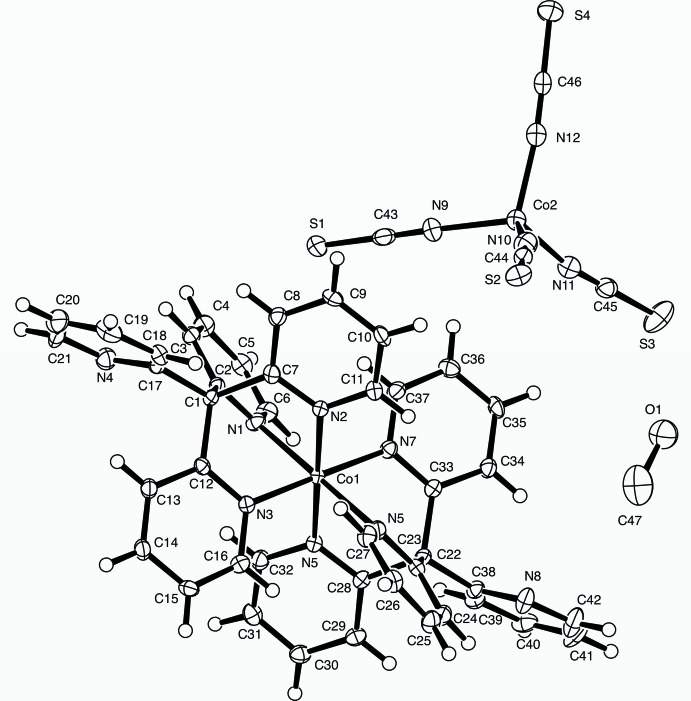
An ellipsoid plot drawing of the title complex, showing 50% probability displacement elipsoids.

## supplementary crystallographic information

### 1. Comment

The coordination chemistry of polypyridyl complexes has been studied extensively due to their coordination ability as ligands, but also due to the possibility to induce strong cooperative effects by intermolecular aromatic interactions which can be utilized in molecular devices and magnetic switches (Hayami *et al.*, 2011; Kalyanasundaram *et al.*, 1998). Among numerous polypyridine ligands tris(pyridin-2-yl)methane, CH(py)_3_, and its derivatives were synthesized and their metal complexes have been studied because of the presence of tripodal π-acceptor ligands. Tetrapyridylmethane would be an especially useful building block for the construction of tetrahedral networks through complexation with transition metal complexes (Matsumoto *et al.*, 2003).

The ligand is tetradentate. However, one of pyridines does not coordinate to the cobalt ion. As a result, [Co{C(py)_4_}]^2+^ (C(py)_4_ = tetrakis(pyridin-2-yl)methane) is mononuclear with the cobolt atom Co(1) coordinated to two C(py)_4_ ligands. The distances of Co(1) – N(2, 3, 6, 7) are shorter than those of Co(1) – N(1, 5), which exhibits a distorted CoN6 octahedron. The average coordination bond Co–N distance in the cation (Co – N 2.029 Å) is shorter than that in the Co^II^ complex of CH(py)_3_ (Co - N 2.109 Å) (Adam *et al.*, 1997). This result suggests that the spin state of [Co{C(py)_4_}_2_]^2+^ is the low spin state. In contrast, the spin state of the Co^II^ ion in [Co{CH(py)_3_}_2_]^2+^ is the high spin state.

There is intermolecular π-π stacking between the pyridine ring in coordinated C(py)_4_ of the cation and NCS ligand of the anion. The length between C(9) and C(45) is 3.500 (5) Å. There are two weak hydrogen-bond-like interactions between methanol solvent molecule and NCS ligands of the anion [O1 – S1 is 3.283 (3) Å, O2 – S4 is 3.170 (2) Å].

### 2. Experimental

A methanol solution (10 ml) of Co(ClO_4_)_2_·6H_2_O (26 mg, 10 mmol) and KSCN (39 mg, 40 mmol) was treated dropwise by cannula transfer with a methanol solution (10 ml) of C(py)_4_ (32 mg, 10 mmol). The reaction mixture was stirred overnight, to give green crystals in 50% yield.

### 3. Refinement

The C-bound hydrogen atoms in the C(py)_4_ were placed at calculated positions, C–H 0.950 Å, and were treated as riding on their parent atoms with *U*_iso_(H) set to 1.2 *U*_eq_(C). The oxygen atom of the methanol solvent molecule was modelled as disordered over three sites with occupation ratio of 8:1:1, and it was refined isotropically. The hydrogen atoms were not included for the occluded methanol molecule due to the fact that they could not be located in the difference map.

### Crystal data

**Table:**

[Co(C_21_H_16_N_4_)_2_][Co(NCS)_4_]·CH_4_O	*F*(000) = 1048.00
*M**_r_* = 1026.96	*D*_x_ = 1.507 Mg m^−^^3^
Monoclinic, *P*2_1_	Mo *K*α radiation, λ = 0.71075 Å
Hall symbol: P 2yb	Cell parameters from 7489 reflections
*a* = 8.903 (3) Å	θ = 3.1–27.5°
*b* = 21.732 (6) Å	µ = 0.97 mm^−^^1^
*c* = 12.335 (4) Å	*T* = 100 K
β = 108.480 (4)°	Platelet, green
*V* = 2263.4 (11) Å^3^	0.70 × 0.20 × 0.10 mm
*Z* = 2	

### Data collection

**Table:**

Rigaku Saturn724 diffractometer	10355 independent reflections
Radiation source: Mo-Ka	9357 reflections with *F*^2^ > 2.0σ(*F*^2^)
Graphite monochromator	*R*_int_ = 0.039
Detector resolution: 7.111 pixels mm^-1^	θ_max_ = 27.5°
ω scans	*h* = −11→11
Absorption correction: multi-scan (*REQAB*; Rigaku, 1998)	*k* = −28→28
*T*_min_ = 0.796, *T*_max_ = 0.908	*l* = −16→16
35095 measured reflections	

### Refinement

**Table:**

Refinement on *F*^2^	Secondary atom site location: difference Fourier map
Least-squares matrix: full	Hydrogen site location: inferred from neighbouring sites
*R*[*F*^2^ > 2σ(*F*^2^)] = 0.034	H-atom parameters constrained
*wR*(*F*^2^) = 0.071	*w* = 1/[σ^2^(*F*_o_^2^) + (0.0341*P*)^2^] where *P* = (*F*_o_^2^ + 2*F*_c_^2^)/3
*S* = 1.01	(Δ/σ)_max_ = 0.001
10355 reflections	Δρ_max_ = 0.49 e Å^−^^3^
596 parameters	Δρ_min_ = −0.35 e Å^−^^3^
4 restraints	Absolute structure: Flack (1983), 5024 Friedel pairs
0 constraints	Absolute structure parameter: −0.008 (8)
Primary atom site location: structure-invariant direct methods	

### Special details

**Table:**

Geometry. ENTER SPECIAL DETAILS OF THE MOLECULAR GEOMETRY
Refinement. Refinement was performed using all reflections. The weighted *R*-factor (*wR*) and goodness of fit (*S*) are based on *F*^2^. *R*-factor (gt) are based on *F*. The threshold expression of *F*^2^ > 2.0 σ(*F*^2^) is used only for calculating *R*-factor (gt).

### Fractional atomic coordinates and isotropic or equivalent isotropic displacement parameters (Å^2^)

**Table:**

	*x*	*y*	*z*	*U*_iso_*/*U*_eq_	Occ. (<1)
Co1	0.92921 (4)	0.267330 (16)	0.12705 (3)	0.01337 (7)	
Co2	0.68958 (4)	−0.017879 (16)	0.32111 (3)	0.02202 (8)	
S1	0.60841 (10)	0.08300 (4)	0.63708 (7)	0.0428 (3)	
S2	0.27520 (8)	−0.00264 (3)	−0.02897 (6)	0.02743 (16)	
S3	1.15687 (8)	0.04825 (3)	0.25418 (7)	0.02932 (16)	
S4	0.80609 (9)	−0.22424 (4)	0.44057 (6)	0.03224 (17)	
O1	0.6953 (4)	0.21625 (13)	−0.2326 (3)	0.0426 (7)*	0.8000
O2	0.553 (3)	0.2545 (11)	−0.4248 (14)	0.0426 (7)*	0.1000
O3	0.568 (3)	0.2338 (10)	−0.2633 (19)	0.0426 (7)*	0.1000
N1	1.1687 (3)	0.23180 (10)	0.17071 (17)	0.0179 (5)	
N2	0.8538 (3)	0.18862 (9)	0.04369 (17)	0.0164 (5)	
N3	0.9621 (3)	0.29708 (9)	−0.01050 (17)	0.0145 (4)	
N4	1.2984 (3)	0.17133 (11)	−0.10782 (19)	0.0239 (5)	
N5	0.6893 (3)	0.30239 (10)	0.07976 (17)	0.0168 (5)	
N6	1.0051 (3)	0.34582 (9)	0.21040 (17)	0.0164 (5)	
N7	0.8943 (3)	0.23901 (9)	0.26477 (17)	0.0160 (5)	
N8	0.5567 (3)	0.37038 (12)	0.35210 (18)	0.0240 (5)	
N9	0.6644 (3)	0.03202 (11)	0.4469 (2)	0.0283 (6)	
N10	0.5046 (3)	−0.01099 (12)	0.1856 (2)	0.0286 (5)	
N11	0.8765 (3)	0.00762 (11)	0.2834 (2)	0.0281 (6)	
N12	0.7249 (3)	−0.10308 (11)	0.3740 (2)	0.0280 (6)	
C1	1.0845 (3)	0.19529 (11)	−0.02558 (19)	0.0130 (5)	
C2	1.2067 (3)	0.19550 (11)	0.0959 (2)	0.0137 (5)	
C3	1.3348 (3)	0.15487 (11)	0.1304 (2)	0.0169 (5)	
C4	1.4287 (3)	0.15497 (13)	0.2436 (3)	0.0222 (6)	
C5	1.3936 (3)	0.19419 (12)	0.3202 (3)	0.0211 (6)	
C6	1.2629 (3)	0.23195 (12)	0.2808 (2)	0.0214 (6)	
C7	0.9443 (3)	0.15873 (12)	−0.00821 (19)	0.0153 (5)	
C8	0.9230 (3)	0.09645 (12)	−0.0321 (2)	0.0181 (6)	
C9	0.7976 (3)	0.06600 (11)	−0.0140 (3)	0.0187 (6)	
C10	0.6939 (3)	0.09815 (12)	0.0288 (3)	0.0200 (6)	
C11	0.7282 (3)	0.15884 (12)	0.0586 (2)	0.0198 (6)	
C12	1.0363 (3)	0.26212 (12)	−0.06783 (19)	0.0142 (5)	
C13	1.0710 (3)	0.28663 (12)	−0.1619 (2)	0.0182 (6)	
C14	1.0271 (3)	0.34621 (12)	−0.1974 (3)	0.0199 (6)	
C15	0.9488 (3)	0.38129 (12)	−0.1389 (3)	0.0195 (6)	
C16	0.9178 (3)	0.35493 (12)	−0.0469 (2)	0.0183 (6)	
C17	1.1438 (3)	0.16544 (11)	−0.1176 (2)	0.0146 (5)	
C18	1.0338 (3)	0.14235 (12)	−0.2142 (2)	0.0183 (6)	
C19	1.0874 (4)	0.12023 (13)	−0.3003 (3)	0.0276 (7)	
C20	1.2429 (4)	0.12323 (13)	−0.2926 (3)	0.0301 (7)	
C21	1.3463 (4)	0.14949 (14)	−0.1948 (3)	0.0270 (7)	
C22	0.7683 (3)	0.34127 (11)	0.2733 (2)	0.0153 (5)	
C23	0.6478 (3)	0.33964 (11)	0.1521 (2)	0.0150 (5)	
C24	0.5154 (3)	0.37739 (12)	0.1138 (3)	0.0202 (6)	
C25	0.4206 (3)	0.37374 (13)	0.0009 (3)	0.0218 (6)	
C26	0.4605 (3)	0.33322 (12)	−0.0721 (3)	0.0209 (6)	
C27	0.5961 (3)	0.29867 (12)	−0.0292 (2)	0.0205 (6)	
C28	0.9117 (3)	0.37688 (11)	0.2577 (2)	0.0143 (5)	
C29	0.9349 (3)	0.43912 (12)	0.2826 (3)	0.0189 (6)	
C30	1.0647 (3)	0.46842 (11)	0.2668 (2)	0.0204 (6)	
C31	1.1695 (3)	0.43477 (12)	0.2289 (3)	0.0207 (6)	
C32	1.1347 (3)	0.37428 (12)	0.1984 (2)	0.0194 (6)	
C33	0.8142 (3)	0.27424 (12)	0.31709 (19)	0.0149 (5)	
C34	0.7701 (3)	0.25079 (11)	0.4075 (2)	0.0187 (6)	
C35	0.8142 (4)	0.19158 (12)	0.4468 (2)	0.0203 (6)	
C36	0.9022 (3)	0.15725 (12)	0.3963 (2)	0.0210 (6)	
C37	0.9392 (3)	0.18219 (12)	0.3049 (3)	0.0201 (6)	
C38	0.7119 (3)	0.37234 (12)	0.3651 (2)	0.0163 (5)	
C39	0.8216 (3)	0.39400 (12)	0.4656 (2)	0.0194 (6)	
C40	0.7700 (4)	0.41589 (12)	0.5518 (3)	0.0244 (6)	
C41	0.6098 (4)	0.41455 (14)	0.5386 (3)	0.0306 (7)	
C42	0.5100 (4)	0.39090 (16)	0.4386 (3)	0.0326 (8)	
C43	0.6394 (3)	0.05312 (12)	0.5246 (3)	0.0242 (6)	
C44	0.4078 (3)	−0.00765 (12)	0.0963 (3)	0.0230 (6)	
C45	0.9930 (4)	0.02487 (12)	0.2702 (3)	0.0248 (6)	
C46	0.7573 (3)	−0.15376 (13)	0.4021 (2)	0.0209 (6)	
C47	0.6522 (4)	0.2642 (3)	−0.3180 (3)	0.0528 (10)	
H1	1.3568	0.1277	0.0771	0.0203*	
H2	1.5172	0.1281	0.2687	0.0266*	
H3	1.4577	0.1952	0.3981	0.0253*	
H4	1.2382	0.2591	0.3331	0.0257*	
H5	0.9949	0.0748	−0.0610	0.0217*	
H6	0.7820	0.0233	−0.0306	0.0225*	
H7	0.6024	0.0790	0.0372	0.0240*	
H8	0.6606	0.1808	0.0911	0.0238*	
H9	1.1249	0.2622	−0.2017	0.0218*	
H10	1.0506	0.3628	−0.2614	0.0239*	
H11	0.9173	0.4223	−0.1615	0.0234*	
H12	0.8626	0.3786	−0.0069	0.0219*	
H13	0.9243	0.1416	−0.2215	0.0220*	
H14	1.0139	0.1025	−0.3664	0.0331*	
H15	1.2794	0.1079	−0.3519	0.0362*	
H16	1.4553	0.1524	−0.1884	0.0325*	
H17	0.4903	0.4054	0.1646	0.0242*	
H18	0.3290	0.3988	−0.0264	0.0262*	
H19	0.3961	0.3293	−0.1496	0.0251*	
H20	0.6248	0.2710	−0.0790	0.0246*	
H21	0.8626	0.4614	0.3101	0.0227*	
H22	1.0813	0.5112	0.2819	0.0245*	
H23	1.2640	0.4530	0.2239	0.0249*	
H24	1.2035	0.3517	0.1681	0.0233*	
H25	0.7097	0.2753	0.4422	0.0224*	
H26	0.7837	0.1751	0.5081	0.0244*	
H27	0.9372	0.1171	0.4234	0.0252*	
H28	0.9989	0.1582	0.2689	0.0242*	
H29	0.9315	0.3936	0.4743	0.0232*	
H30	0.8434	0.4318	0.6199	0.0293*	
H31	0.5704	0.4295	0.5968	0.0367*	
H32	0.4003	0.3889	0.4302	0.0391*	

### Atomic displacement parameters (Å^2^)

**Table:**

	*U*^11^	*U*^22^	*U*^33^	*U*^12^	*U*^13^	*U*^23^
Co1	0.01431 (15)	0.01349 (15)	0.01381 (15)	0.00066 (13)	0.00660 (12)	0.00050 (13)
Co2	0.02042 (17)	0.02224 (18)	0.02485 (19)	0.00059 (15)	0.00924 (15)	0.00178 (15)
S1	0.0341 (5)	0.0537 (6)	0.0386 (5)	0.0079 (4)	0.0086 (4)	−0.0209 (4)
S2	0.0271 (4)	0.0249 (4)	0.0274 (4)	0.0032 (3)	0.0044 (3)	−0.0035 (3)
S3	0.0276 (4)	0.0223 (4)	0.0438 (5)	0.0006 (3)	0.0195 (4)	0.0056 (3)
S4	0.0371 (4)	0.0267 (4)	0.0340 (4)	0.0072 (4)	0.0129 (4)	0.0088 (4)
N1	0.0214 (11)	0.0180 (11)	0.0143 (10)	0.0037 (9)	0.0057 (9)	−0.0010 (9)
N2	0.0177 (11)	0.0142 (11)	0.0199 (11)	0.0022 (9)	0.0099 (9)	0.0026 (9)
N3	0.0140 (10)	0.0155 (11)	0.0145 (10)	−0.0005 (8)	0.0050 (9)	0.0005 (8)
N4	0.0230 (13)	0.0260 (13)	0.0238 (12)	0.0024 (10)	0.0089 (11)	0.0034 (10)
N5	0.0156 (11)	0.0196 (11)	0.0162 (11)	0.0004 (9)	0.0065 (9)	0.0024 (9)
N6	0.0164 (11)	0.0168 (11)	0.0178 (11)	0.0010 (9)	0.0080 (9)	0.0005 (9)
N7	0.0162 (11)	0.0178 (11)	0.0141 (10)	0.0018 (9)	0.0048 (9)	−0.0003 (9)
N8	0.0176 (11)	0.0370 (14)	0.0187 (12)	0.0053 (10)	0.0076 (10)	−0.0004 (10)
N9	0.0297 (13)	0.0243 (13)	0.0326 (14)	−0.0003 (10)	0.0124 (12)	0.0007 (11)
N10	0.0261 (12)	0.0303 (14)	0.0295 (13)	0.0002 (11)	0.0086 (11)	−0.0016 (11)
N11	0.0268 (13)	0.0267 (13)	0.0357 (14)	−0.0020 (10)	0.0168 (11)	0.0036 (11)
N12	0.0282 (13)	0.0286 (14)	0.0322 (14)	0.0013 (11)	0.0165 (11)	0.0028 (11)
C1	0.0129 (12)	0.0145 (12)	0.0120 (12)	0.0015 (10)	0.0044 (10)	−0.0006 (9)
C2	0.0149 (12)	0.0135 (12)	0.0136 (12)	−0.0012 (10)	0.0059 (10)	−0.0011 (9)
C3	0.0145 (12)	0.0180 (13)	0.0182 (13)	0.0010 (10)	0.0049 (10)	0.0021 (10)
C4	0.0165 (13)	0.0256 (15)	0.0224 (14)	0.0019 (11)	0.0032 (11)	0.0034 (11)
C5	0.0197 (14)	0.0243 (14)	0.0167 (13)	−0.0038 (11)	0.0020 (11)	0.0012 (11)
C6	0.0267 (15)	0.0208 (14)	0.0162 (13)	−0.0010 (11)	0.0060 (11)	−0.0038 (11)
C7	0.0155 (12)	0.0204 (13)	0.0092 (11)	0.0011 (10)	0.0028 (10)	0.0025 (10)
C8	0.0202 (13)	0.0191 (13)	0.0165 (13)	0.0021 (11)	0.0079 (11)	−0.0003 (10)
C9	0.0225 (14)	0.0126 (13)	0.0203 (13)	−0.0044 (10)	0.0057 (11)	−0.0004 (10)
C10	0.0184 (13)	0.0203 (14)	0.0229 (14)	−0.0044 (11)	0.0088 (11)	0.0045 (11)
C11	0.0199 (13)	0.0203 (14)	0.0224 (14)	0.0000 (11)	0.0111 (11)	0.0023 (11)
C12	0.0117 (11)	0.0149 (12)	0.0157 (11)	−0.0024 (10)	0.0038 (9)	−0.0000 (10)
C13	0.0175 (13)	0.0209 (14)	0.0182 (12)	0.0006 (10)	0.0085 (10)	0.0017 (10)
C14	0.0189 (13)	0.0237 (14)	0.0196 (13)	0.0003 (11)	0.0096 (11)	0.0037 (11)
C15	0.0219 (13)	0.0136 (13)	0.0232 (14)	0.0013 (11)	0.0073 (12)	0.0039 (10)
C16	0.0178 (13)	0.0177 (13)	0.0191 (13)	0.0022 (10)	0.0053 (11)	0.0000 (10)
C17	0.0186 (13)	0.0123 (12)	0.0145 (12)	0.0019 (10)	0.0073 (10)	0.0012 (10)
C18	0.0185 (13)	0.0203 (13)	0.0164 (13)	−0.0028 (11)	0.0058 (11)	0.0025 (10)
C19	0.0411 (18)	0.0237 (15)	0.0149 (14)	−0.0058 (13)	0.0046 (13)	0.0002 (11)
C20	0.0466 (18)	0.0293 (16)	0.0208 (15)	0.0075 (14)	0.0194 (14)	0.0022 (12)
C21	0.0238 (15)	0.0350 (17)	0.0284 (15)	0.0129 (13)	0.0168 (13)	0.0082 (13)
C22	0.0172 (13)	0.0157 (13)	0.0145 (12)	0.0021 (10)	0.0071 (10)	−0.0007 (10)
C23	0.0159 (12)	0.0145 (12)	0.0150 (12)	−0.0022 (10)	0.0057 (10)	0.0015 (10)
C24	0.0185 (13)	0.0233 (14)	0.0207 (13)	0.0035 (11)	0.0091 (11)	0.0017 (11)
C25	0.0153 (13)	0.0294 (15)	0.0200 (13)	0.0043 (11)	0.0046 (11)	0.0048 (11)
C26	0.0170 (13)	0.0260 (15)	0.0182 (13)	−0.0026 (11)	0.0032 (11)	0.0002 (11)
C27	0.0211 (14)	0.0240 (14)	0.0163 (13)	−0.0020 (11)	0.0059 (11)	−0.0040 (11)
C28	0.0120 (11)	0.0175 (13)	0.0118 (11)	0.0019 (10)	0.0014 (10)	−0.0006 (10)
C29	0.0184 (13)	0.0183 (13)	0.0184 (13)	0.0020 (11)	0.0035 (11)	−0.0013 (10)
C30	0.0214 (13)	0.0164 (14)	0.0225 (14)	−0.0013 (10)	0.0055 (11)	−0.0024 (10)
C31	0.0164 (13)	0.0245 (15)	0.0199 (14)	−0.0033 (11)	0.0037 (11)	0.0014 (11)
C32	0.0168 (12)	0.0218 (14)	0.0205 (13)	0.0014 (11)	0.0071 (11)	0.0010 (11)
C33	0.0125 (11)	0.0173 (13)	0.0137 (11)	0.0003 (10)	0.0026 (9)	−0.0014 (10)
C34	0.0201 (13)	0.0209 (14)	0.0173 (13)	0.0016 (10)	0.0092 (11)	−0.0004 (10)
C35	0.0266 (15)	0.0237 (14)	0.0137 (13)	−0.0033 (12)	0.0106 (12)	0.0032 (11)
C36	0.0243 (14)	0.0190 (14)	0.0185 (13)	0.0012 (11)	0.0050 (11)	0.0034 (11)
C37	0.0243 (14)	0.0193 (14)	0.0193 (14)	0.0054 (11)	0.0105 (12)	0.0012 (10)
C38	0.0189 (13)	0.0171 (13)	0.0137 (12)	0.0027 (11)	0.0063 (11)	−0.0006 (10)
C39	0.0188 (13)	0.0193 (14)	0.0190 (13)	0.0017 (11)	0.0044 (11)	0.0005 (10)
C40	0.0345 (16)	0.0198 (14)	0.0162 (13)	0.0061 (12)	0.0040 (12)	−0.0023 (11)
C41	0.0351 (17)	0.0390 (18)	0.0204 (15)	0.0206 (14)	0.0127 (13)	0.0004 (13)
C42	0.0238 (15)	0.053 (2)	0.0246 (16)	0.0135 (14)	0.0127 (13)	0.0048 (14)
C43	0.0203 (14)	0.0198 (14)	0.0320 (16)	−0.0010 (11)	0.0076 (12)	0.0006 (12)
C44	0.0248 (14)	0.0177 (14)	0.0315 (15)	−0.0006 (11)	0.0160 (12)	−0.0013 (11)
C45	0.0306 (16)	0.0167 (13)	0.0279 (15)	0.0071 (12)	0.0105 (13)	0.0020 (11)
C46	0.0172 (13)	0.0289 (16)	0.0175 (13)	−0.0014 (11)	0.0070 (11)	0.0000 (11)
C47	0.046 (2)	0.080 (3)	0.0289 (17)	−0.009 (3)	0.0069 (15)	−0.003 (2)

### Geometric parameters (Å, º)

**Table:**

Co1—N1	2.169 (3)	C18—C19	1.382 (5)
Co1—N2	1.999 (2)	C19—C20	1.358 (5)
Co1—N3	1.923 (3)	C20—C21	1.387 (4)
Co1—N5	2.166 (2)	C22—C23	1.539 (3)
Co1—N6	1.996 (2)	C22—C28	1.557 (4)
Co1—N7	1.923 (3)	C22—C33	1.562 (4)
Co2—N9	1.964 (3)	C22—C38	1.533 (4)
Co2—N10	1.945 (2)	C23—C24	1.390 (4)
Co2—N11	1.945 (3)	C24—C25	1.384 (4)
Co2—N12	1.955 (3)	C25—C26	1.383 (5)
S1—C43	1.632 (4)	C26—C27	1.377 (4)
S2—C44	1.623 (3)	C28—C29	1.388 (4)
S3—C45	1.615 (4)	C29—C30	1.386 (4)
S4—C46	1.621 (3)	C30—C31	1.378 (4)
O1—O3	1.14 (3)	C31—C32	1.375 (4)
O1—C47	1.444 (5)	C33—C34	1.391 (4)
O2—C47	1.350 (16)	C34—C35	1.387 (4)
O3—C47	1.33 (3)	C35—C36	1.367 (5)
N1—C2	1.336 (4)	C36—C37	1.381 (5)
N1—C6	1.351 (3)	C38—C39	1.394 (4)
N2—C7	1.344 (4)	C39—C40	1.371 (5)
N2—C11	1.354 (4)	C40—C41	1.383 (5)
N3—C12	1.345 (4)	C41—C42	1.371 (4)
N3—C16	1.351 (4)	C3—H1	0.950
N4—C17	1.349 (4)	C4—H2	0.950
N4—C21	1.360 (5)	C5—H3	0.950
N5—C23	1.341 (4)	C6—H4	0.950
N5—C27	1.340 (3)	C8—H5	0.950
N6—C28	1.339 (4)	C9—H6	0.950
N6—C32	1.358 (4)	C10—H7	0.950
N7—C33	1.342 (4)	C11—H8	0.950
N7—C37	1.343 (4)	C13—H9	0.950
N8—C38	1.341 (4)	C14—H10	0.950
N8—C42	1.339 (5)	C15—H11	0.950
N9—C43	1.146 (5)	C16—H12	0.950
N10—C44	1.166 (3)	C18—H13	0.950
N11—C45	1.162 (4)	C19—H14	0.950
N12—C46	1.163 (4)	C20—H15	0.950
C1—C2	1.548 (3)	C21—H16	0.950
C1—C7	1.551 (4)	C24—H17	0.950
C1—C12	1.557 (4)	C25—H18	0.950
C1—C17	1.539 (4)	C26—H19	0.950
C2—C3	1.397 (4)	C27—H20	0.950
C3—C4	1.382 (4)	C29—H21	0.950
C4—C5	1.380 (5)	C30—H22	0.950
C5—C6	1.380 (4)	C31—H23	0.950
C7—C8	1.385 (4)	C32—H24	0.950
C8—C9	1.376 (4)	C34—H25	0.950
C9—C10	1.388 (5)	C35—H26	0.950
C10—C11	1.377 (4)	C36—H27	0.950
C12—C13	1.397 (4)	C37—H28	0.950
C13—C14	1.383 (4)	C39—H29	0.950
C14—C15	1.381 (5)	C40—H30	0.950
C15—C16	1.376 (4)	C41—H31	0.950
C17—C18	1.375 (4)	C42—H32	0.950
			
Co1···C1	3.091 (3)	H27···H28	2.3218
Co1···C22	3.087 (3)	H29···H30	2.3292
N1···C4	2.763 (4)	H30···H31	2.3560
N1···C7	2.930 (3)	H31···H32	2.3059
N1···C12	2.878 (3)	Co2···H2^iv^	3.4945
N1···C32	3.140 (4)	Co2···H31^vii^	3.0294
N1···C37	3.197 (4)	S1···H13^i^	3.0767
N2···C2	3.004 (4)	S1···H15^xiii^	3.0240
N2···C9	2.763 (4)	S1···H16^xiii^	3.2595
N2···C12	2.916 (4)	S1···H22^xii^	3.0523
N2···C27	3.238 (4)	S1···H23^xii^	3.3128
N2···C37	3.070 (4)	S1···H26	3.2479
N3···C2	3.086 (3)	S2···H1^iv^	3.1080
N3···C7	3.011 (4)	S2···H5^iv^	2.9307
N3···C14	2.763 (4)	S2···H7	3.2842
N3···C27	3.193 (4)	S2···H12^ii^	2.9513
N3···C32	3.051 (3)	S2···H21^ii^	3.3827
N4···C2	2.926 (4)	S3···H11^xiv^	2.9582
N4···C3	2.875 (4)	S3···H28	2.8068
N4···C12	3.211 (4)	S3···H30^xii^	2.9702
N4···C13	3.157 (4)	S4···H4^xii^	2.9633
N4···C19	2.752 (4)	S4···H9^xiv^	3.2081
N5···C11	3.159 (4)	S4···H10^xiv^	3.4464
N5···C16	3.146 (4)	S4···H29^xii^	3.4004
N5···C25	2.757 (4)	O1···H13	2.5745
N5···C28	2.933 (3)	O1···H16^iv^	2.7439
N5···C33	2.849 (3)	O1···H20	2.4807
N6···C6	3.299 (4)	O2···H3^vi^	2.4450
N6···C16	3.025 (4)	O2···H4^vi^	3.3876
N6···C23	3.033 (4)	O2···H25^iii^	2.5093
N6···C30	2.762 (4)	O2···H26^iii^	2.9937
N6···C33	2.908 (4)	O2···H32^iii^	3.4697
N7···C6	3.228 (4)	O3···H16^iv^	2.3635
N7···C11	3.051 (3)	O3···H19	3.1596
N7···C23	3.098 (3)	O3···H20	2.3123
N7···C28	3.003 (4)	N4···H7^ix^	3.3888
N7···C35	2.762 (4)	N4···H8^ix^	3.3831
N8···C23	2.908 (4)	N4···H20^ix^	3.5510
N8···C24	2.848 (4)	N8···H23^iv^	3.1468
N8···C33	3.231 (4)	N8···H24^iv^	3.2677
N8···C34	3.164 (4)	N9···H2^iv^	3.0143
N8···C40	2.773 (4)	N9···H14^i^	3.5768
C2···C5	2.739 (4)	N9···H22^xii^	3.4229
C2···C8	3.313 (4)	N9···H26	3.2955
C3···C6	2.722 (4)	N9···H27	3.1390
C3···C7	3.354 (4)	N9···H31^vii^	2.9846
C3···C17	3.001 (4)	N9···H32^vii^	3.5847
C6···C32	3.342 (4)	N10···H1^iv^	3.3897
C6···C37	3.179 (5)	N10···H2^iv^	3.1826
C7···C10	2.749 (4)	N10···H7	2.9896
C7···C18	2.915 (4)	N10···H18^ii^	3.4258
C8···C11	2.707 (4)	N10···H31^vii^	3.2347
C8···C17	2.921 (4)	N11···H7	3.5896
C8···C18	2.900 (5)	N11···H10^xiv^	3.2414
C11···C27	3.314 (4)	N11···H11^xiv^	3.2891
C11···C37	3.066 (4)	N11···H27	2.8889
C12···C15	2.766 (4)	N11···H28	3.4706
C12···C18	3.164 (4)	N11···H30^xii^	2.9110
C13···C16	2.702 (4)	N12···H10^xiv^	2.8686
C13···C17	2.726 (4)	N12···H19^ii^	3.0149
C13···C18	3.197 (4)	N12···H29^xii^	3.0394
C16···C27	3.184 (4)	N12···H31^vii^	2.8535
C16···C32	3.059 (4)	N12···H32^vii^	2.9685
C17···C20	2.736 (5)	C3···H7^ix^	3.3847
C18···C21	2.721 (5)	C3···H8^ix^	3.1406
C23···C26	2.743 (4)	C4···H8^ix^	3.2538
C23···C29	3.345 (4)	C5···H25^ix^	3.2571
C23···C34	3.558 (4)	C5···H26^ix^	3.5358
C24···C27	2.713 (4)	C8···H22^xiv^	3.5854
C24···C28	3.410 (4)	C9···H23^xiv^	3.4830
C24···C38	3.041 (4)	C10···H1^iv^	3.3019
C28···C31	2.737 (4)	C10···H16^iv^	3.0766
C28···C39	2.943 (4)	C11···H1^iv^	3.4519
C29···C32	2.719 (4)	C11···H16^iv^	3.2416
C29···C38	2.893 (4)	C13···H18^ix^	3.4035
C29···C39	2.919 (5)	C13···H19^ix^	2.9967
C33···C36	2.747 (4)	C14···H18^ix^	3.0639
C33···C39	3.171 (4)	C14···H19^ix^	3.1690
C34···C37	2.701 (5)	C14···H30^iii^	2.9791
C34···C38	2.711 (4)	C15···H18^ix^	3.2480
C34···C39	3.194 (4)	C15···H30^iii^	3.0277
C38···C41	2.735 (5)	C18···H22^xiv^	3.0549
C39···C42	2.688 (5)	C18···H26^iii^	3.5166
S1···O1^i^	3.283 (3)	C19···H21^xiv^	3.4870
S1···O3^i^	3.56 (3)	C19···H22^xiv^	2.8533
S2···C24^ii^	3.550 (3)	C19···H26^iii^	3.2047
S2···C29^ii^	3.343 (3)	C19···H27^iii^	3.2433
S2···C30^ii^	3.540 (3)	C21···H7^ix^	3.4058
S3···C3	3.426 (3)	C21···H20^ix^	3.5940
S3···C4	3.384 (3)	C24···H23^iv^	3.3883
S4···O2^ii^	3.17 (2)	C24···H24^iv^	3.1063
S4···O3^ii^	3.48 (2)	C25···H24^iv^	3.2782
O1···S1^iii^	3.283 (3)	C26···H9^iv^	3.2911
O1···N2	3.304 (4)	C30···H5^xv^	3.3489
O1···N3	3.480 (4)	C30···H14^xv^	3.3230
O1···C7	3.201 (4)	C30···H32^ix^	3.4865
O1···C12	3.229 (4)	C31···H6^xv^	3.2473
O1···C13	3.526 (4)	C31···H17^ix^	3.2599
O1···C18	3.358 (5)	C31···H32^ix^	2.8544
O1···C21^iv^	3.590 (5)	C32···H17^ix^	3.3900
O1···C27	3.418 (5)	C32···H32^ix^	3.0956
O2···S4^v^	3.17 (2)	C34···H2^iv^	3.5588
O2···C5^vi^	3.286 (18)	C34···H3^iv^	3.0008
O2···C34^iii^	3.25 (3)	C35···H2^iv^	3.1687
O2···C35^iii^	3.48 (3)	C35···H3^iv^	3.0382
O2···C41^iii^	3.56 (3)	C35···H14^i^	3.1011
O2···C42^iii^	3.37 (3)	C36···H2^iv^	3.3482
O2···C46^v^	3.49 (3)	C36···H14^i^	3.0210
O3···S1^iii^	3.56 (3)	C39···H10^i^	3.4058
O3···S4^v^	3.48 (2)	C40···H10^i^	3.0376
O3···C21^iv^	3.00 (3)	C40···H11^i^	3.3616
O3···C26	3.55 (3)	C42···H23^iv^	3.1559
O3···C27	3.15 (3)	C43···H2^iv^	3.4102
N2···O1	3.304 (4)	C43···H14^i^	3.3521
N3···O1	3.480 (4)	C43···H22^xii^	2.9908
N8···C31^iv^	3.582 (4)	C43···H26	2.9811
N9···C41^vii^	3.575 (4)	C43···H27	3.5527
N11···C36	3.516 (4)	C43···H31^vii^	3.3429
N12···C41^vii^	3.496 (5)	C44···H1^iv^	2.9742
N12···C42^vii^	3.577 (5)	C44···H2^iv^	3.5865
C3···S3	3.426 (3)	C44···H7	2.8061
C4···S3	3.384 (3)	C44···H11^ii^	3.5796
C5···O2^viii^	3.286 (18)	C44···H12^ii^	3.3826
C5···C34^ix^	3.409 (4)	C44···H18^ii^	3.4128
C5···C35^ix^	3.572 (4)	C45···H6	3.5878
C7···O1	3.201 (4)	C45···H10^xiv^	3.5405
C9···C45	3.499 (4)	C45···H11^xiv^	2.8421
C12···O1	3.229 (4)	C45···H27	2.9043
C13···O1	3.526 (4)	C45···H28	2.8971
C13···C26^ix^	3.441 (4)	C45···H30^xii^	2.6080
C14···C40^iii^	3.551 (4)	C46···H9^xiv^	3.4908
C18···O1	3.358 (5)	C46···H10^xiv^	2.8188
C21···O1^ix^	3.590 (5)	C46···H19^ii^	3.0011
C21···O3^ix^	3.00 (3)	C46···H29^xii^	2.8950
C24···S2^v^	3.550 (3)	C46···H31^vii^	3.4367
C25···C44^v^	3.402 (5)	C46···H32^vii^	2.9911
C26···O3	3.55 (3)	C47···H13	3.5427
C26···C13^iv^	3.441 (4)	C47···H20	3.0379
C27···O1	3.418 (5)	C47···H25^iii^	3.1664
C27···O3	3.15 (3)	C47···H26^iii^	3.3628
C29···S2^v^	3.343 (3)	H1···S2^ix^	3.1080
C30···S2^v^	3.540 (3)	H1···N10^ix^	3.3897
C30···C43^x^	3.556 (4)	H1···C10^ix^	3.3019
C31···N8^ix^	3.582 (4)	H1···C11^ix^	3.4519
C31···C42^ix^	3.438 (4)	H1···C44^ix^	2.9742
C34···O2^i^	3.25 (3)	H1···H7^ix^	2.6120
C34···C5^iv^	3.409 (4)	H1···H8^ix^	2.8949
C35···O2^i^	3.48 (3)	H2···Co2^ix^	3.4945
C35···C5^iv^	3.572 (4)	H2···N9^ix^	3.0143
C36···N11	3.516 (4)	H2···N10^ix^	3.1826
C36···C45	3.485 (4)	H2···C34^ix^	3.5588
C37···C45	3.497 (4)	H2···C35^ix^	3.1687
C40···C14^i^	3.551 (4)	H2···C36^ix^	3.3482
C40···C45^x^	3.456 (4)	H2···C43^ix^	3.4102
C41···O2^i^	3.56 (3)	H2···C44^ix^	3.5865
C41···N9^xi^	3.575 (4)	H2···H7^ix^	3.3532
C41···N12^xi^	3.496 (5)	H2···H8^ix^	3.0853
C42···O2^i^	3.37 (3)	H2···H26^ix^	3.3092
C42···N12^xi^	3.577 (5)	H3···O2^viii^	2.4450
C42···C31^iv^	3.438 (4)	H3···C34^ix^	3.0008
C43···C30^xii^	3.556 (4)	H3···C35^ix^	3.0382
C44···C25^ii^	3.402 (5)	H3···H25^ix^	2.7546
C45···C9	3.499 (4)	H3···H26^ix^	2.8206
C45···C36	3.485 (4)	H4···S4^x^	2.9633
C45···C37	3.497 (4)	H4···O2^viii^	3.3876
C45···C40^xii^	3.456 (4)	H4···H32^ix^	3.2220
C46···O2^ii^	3.49 (3)	H5···S2^ix^	2.9307
Co1···H4	3.1025	H5···C30^xiv^	3.3489
Co1···H8	2.9625	H5···H22^xiv^	2.9370
Co1···H12	2.8813	H6···C31^xiv^	3.2473
Co1···H20	3.0737	H6···C45	3.5878
Co1···H24	2.9659	H6···H17^ii^	3.5554
Co1···H28	2.8961	H6···H18^ii^	3.0379
N1···H1	3.2383	H6···H23^xiv^	2.7512
N1···H3	3.2480	H7···S2	3.2842
N1···H24	2.6251	H7···N4^iv^	3.3888
N1···H28	2.7338	H7···N10	2.9896
N2···H5	3.2238	H7···N11	3.5896
N2···H7	3.2535	H7···C3^iv^	3.3847
N2···H20	2.7752	H7···C21^iv^	3.4058
N2···H28	2.7474	H7···C44	2.8061
N3···H9	3.2254	H7···H1^iv^	2.6120
N3···H11	3.2509	H7···H2^iv^	3.3532
N3···H20	2.9064	H7···H16^iv^	3.1129
N3···H24	2.8066	H8···N4^iv^	3.3831
N4···H1	2.3702	H8···C3^iv^	3.1406
N4···H9	2.5484	H8···C4^iv^	3.2538
N4···H13	3.2425	H8···H1^iv^	2.8949
N4···H15	3.2664	H8···H2^iv^	3.0853
N5···H8	2.6624	H8···H16^iv^	3.4072
N5···H12	2.7027	H9···S4^xv^	3.2081
N5···H17	3.2285	H9···C26^ix^	3.2911
N5···H19	3.2372	H9···C46^xv^	3.4908
N6···H4	2.8561	H9···H19^ix^	2.7168
N6···H12	2.6726	H10···S4^xv^	3.4464
N6···H21	3.2278	H10···N11^xv^	3.2414
N6···H23	3.2448	H10···N12^xv^	2.8686
N7···H4	2.9398	H10···C39^iii^	3.4058
N7···H8	2.7734	H10···C40^iii^	3.0376
N7···H25	3.2237	H10···C45^xv^	3.5405
N7···H27	3.2427	H10···C46^xv^	2.8188
N8···H17	2.3257	H10···H18^ix^	3.2565
N8···H21	3.5351	H10···H19^ix^	3.0334
N8···H25	2.5302	H10···H29^iii^	3.1637
N8···H29	3.2363	H10···H30^iii^	2.4658
N8···H31	3.2463	H11···S3^xv^	2.9582
C1···H1	2.7737	H11···N11^xv^	3.2891
C1···H5	2.7318	H11···C40^iii^	3.3616
C1···H9	2.7315	H11···C44^v^	3.5796
C1···H13	2.6547	H11···C45^xv^	2.8421
C2···H2	3.2542	H11···H18^ix^	3.5495
C2···H4	3.1661	H11···H30^iii^	2.5731
C2···H5	3.4453	H11···H31^iii^	3.5518
C2···H24	3.5112	H12···S2^v^	2.9513
C2···H28	3.3371	H12···C44^v^	3.3826
C3···H3	3.2524	H13···S1^iii^	3.0767
C4···H4	3.2248	H13···O1	2.5745
C5···H1	3.2511	H13···C47	3.5427
C5···H28	3.4571	H13···H22^xiv^	2.9279
C6···H2	3.2332	H13···H26^iii^	3.2528
C6···H24	2.9183	H14···N9^iii^	3.5768
C6···H28	2.8107	H14···C30^xiv^	3.3230
C7···H1	3.5500	H14···C35^iii^	3.1011
C7···H6	3.2507	H14···C36^iii^	3.0210
C7···H8	3.1727	H14···C43^iii^	3.3521
C7···H13	2.6061	H14···H21^xiv^	3.2578
C7···H28	3.2964	H14···H22^xiv^	2.5116
C8···H7	3.2517	H14···H26^iii^	2.6600
C8···H13	2.5365	H14···H27^iii^	2.4833
C9···H8	3.2235	H15···S1^xvi^	3.0240
C9···H13	3.5148	H15···H21^xiv^	3.5234
C10···H5	3.2480	H15···H27^iii^	3.4133
C10···H28	3.5703	H16···S1^xvi^	3.2595
C11···H6	3.2318	H16···O1^ix^	2.7439
C11···H20	2.9469	H16···O3^ix^	2.3635
C11···H28	2.9282	H16···C10^ix^	3.0766
C12···H10	3.2715	H16···C11^ix^	3.2416
C12···H12	3.1777	H16···H7^ix^	3.1129
C12···H13	3.2008	H16···H8^ix^	3.4072
C12···H24	3.4243	H16···H20^ix^	3.0753
C13···H11	3.2519	H17···C31^iv^	3.2599
C13···H13	3.4011	H17···C32^iv^	3.3900
C14···H12	3.2167	H17···H6^v^	3.5555
C15···H9	3.2441	H17···H23^iv^	2.5683
C16···H10	3.2276	H17···H24^iv^	2.8200
C16···H20	3.1036	H18···N10^v^	3.4258
C16···H24	3.0377	H18···C13^iv^	3.4035
C17···H1	2.6738	H18···C14^iv^	3.0639
C17···H5	2.5902	H18···C15^iv^	3.2480
C17···H9	2.3278	H18···C44^v^	3.4128
C17···H14	3.2210	H18···H6^v^	3.0379
C17···H16	3.1736	H18···H10^iv^	3.2565
C18···H5	2.5015	H18···H11^iv^	3.5495
C18···H9	2.7186	H18···H23^iv^	3.5185
C18···H15	3.2532	H18···H24^iv^	3.1224
C19···H5	3.4506	H19···O3	3.1596
C19···H9	3.2950	H19···N12^v^	3.0149
C19···H16	3.2023	H19···C13^iv^	2.9967
C20···H9	3.4972	H19···C14^iv^	3.1690
C20···H13	3.2483	H19···C46^v^	3.0011
C21···H1	3.3587	H19···H9^iv^	2.7168
C21···H9	3.1288	H19···H10^iv^	3.0334
C21···H14	3.2105	H20···O1	2.4807
C22···H17	2.7858	H20···O3	2.3123
C22···H21	2.7362	H20···N4^iv^	3.5510
C22···H25	2.7125	H20···C21^iv^	3.5940
C22···H29	2.6954	H20···C47	3.0379
C23···H8	3.5426	H20···H16^iv^	3.0753
C23···H12	3.2551	H21···S2^v^	3.3827
C23···H18	3.2556	H21···C19^xv^	3.4870
C23···H20	3.1663	H21···H14^xv^	3.2578
C23···H21	3.4778	H21···H15^xv^	3.5234
C24···H19	3.2537	H22···S1^x^	3.0523
C25···H20	3.2236	H22···N9^x^	3.4229
C26···H12	3.5506	H22···C8^xv^	3.5854
C26···H17	3.2502	H22···C18^xv^	3.0549
C27···H8	2.9243	H22···C19^xv^	2.8533
C27···H12	2.8815	H22···C43^x^	2.9908
C27···H18	3.2327	H22···H5^xv^	2.9370
C28···H12	3.1526	H22···H13^xv^	2.9279
C28···H22	3.2557	H22···H14^xv^	2.5116
C28···H24	3.1765	H23···S1^x^	3.3128
C28···H29	2.6456	H23···N8^ix^	3.1468
C29···H23	3.2472	H23···C9^xv^	3.4830
C29···H29	2.5721	H23···C24^ix^	3.3883
C30···H24	3.2247	H23···C42^ix^	3.1559
C30···H29	3.5389	H23···H6^xv^	2.7512
C31···H12	3.5185	H23···H17^ix^	2.5683
C31···H21	3.2460	H23···H18^ix^	3.5185
C32···H4	2.9833	H23···H32^ix^	2.8167
C32···H12	2.8986	H24···N8^ix^	3.2677
C32···H22	3.2321	H24···C24^ix^	3.1063
C33···H8	3.3689	H24···C25^ix^	3.2782
C33···H26	3.2664	H24···H17^ix^	2.8200
C33···H28	3.1687	H24···H18^ix^	3.1224
C33···H29	3.2082	H24···H32^ix^	3.2542
C34···H27	3.2409	H25···O2^i^	2.5093
C34···H29	3.4095	H25···C5^iv^	3.2571
C35···H28	3.2149	H25···C47^i^	3.1664
C36···H25	3.2336	H25···H3^iv^	2.7546
C37···H4	3.0689	H26···S1	3.2479
C37···H8	2.9934	H26···O2^i^	2.9937
C37···H26	3.2280	H26···N9	3.2955
C38···H17	2.7263	H26···C5^iv^	3.5358
C38···H21	2.5662	H26···C18^i^	3.5166
C38···H25	2.3159	H26···C19^i^	3.2047
C38···H30	3.2526	H26···C43	2.9811
C38···H32	3.1437	H26···C47^i^	3.3628
C39···H21	2.5305	H26···H2^iv^	3.3092
C39···H25	2.7474	H26···H3^iv^	2.8206
C39···H31	3.2417	H26···H13^i^	3.2528
C40···H21	3.4799	H26···H14^i^	2.6600
C40···H25	3.3154	H27···N9	3.1390
C40···H32	3.2088	H27···N11	2.8889
C41···H25	3.4688	H27···C19^i^	3.2433
C41···H29	3.2373	H27···C43	3.5527
C42···H17	3.3432	H27···C45	2.9043
C42···H25	3.0698	H27···H14^i^	2.4833
C42···H30	3.2229	H27···H15^i^	3.4133
H1···H2	2.3421	H28···S3	2.8068
H1···H5	3.3354	H28···N11	3.4706
H2···H3	2.3429	H28···C45	2.8971
H3···H4	2.3237	H29···S4^x^	3.4004
H4···H24	2.8079	H29···N12^x^	3.0394
H4···H28	2.9855	H29···C46^x^	2.8950
H5···H6	2.3314	H29···H10^i^	3.1637
H5···H13	2.3731	H30···S3^x^	2.9702
H6···H7	2.3591	H30···N11^x^	2.9110
H7···H8	2.3221	H30···C14^i^	2.9791
H8···H20	2.8150	H30···C15^i^	3.0277
H8···H28	3.1518	H30···C45^x^	2.6080
H9···H10	2.3350	H30···H10^i^	2.4658
H9···H13	3.1376	H30···H11^i^	2.5731
H10···H11	2.3511	H31···Co2^xi^	3.0294
H11···H12	2.3174	H31···N9^xi^	2.9846
H12···H20	3.0885	H31···N10^xi^	3.2347
H12···H24	3.1716	H31···N12^xi^	2.8535
H13···H14	2.3367	H31···C43^xi^	3.3429
H14···H15	2.3155	H31···C46^xi^	3.4367
H15···H16	2.3350	H31···H11^i^	3.5518
H17···H18	2.3429	H32···O2^i^	3.4697
H17···H21	3.4522	H32···N9^xi^	3.5847
H18···H19	2.3505	H32···N12^xi^	2.9685
H19···H20	2.3203	H32···C30^iv^	3.4865
H21···H22	2.3470	H32···C31^iv^	2.8544
H21···H29	2.4210	H32···C32^iv^	3.0956
H22···H23	2.3439	H32···C46^xi^	2.9911
H23···H24	2.3195	H32···H4^iv^	3.2220
H25···H26	2.3440	H32···H23^iv^	2.8167
H25···H29	3.1889	H32···H24^iv^	3.2542
H26···H27	2.3348		
			
N1—Co1—N2	88.07 (8)	N5—C27—C26	122.8 (3)
N1—Co1—N3	85.76 (9)	N6—C28—C22	116.7 (2)
N1—Co1—N5	178.82 (9)	N6—C28—C29	121.2 (3)
N1—Co1—N6	91.77 (8)	C22—C28—C29	121.8 (3)
N1—Co1—N7	95.06 (9)	C28—C29—C30	119.3 (3)
N2—Co1—N3	87.37 (9)	C29—C30—C31	119.2 (3)
N2—Co1—N5	91.20 (8)	C30—C31—C32	118.8 (3)
N2—Co1—N6	179.85 (9)	N6—C32—C31	122.1 (3)
N2—Co1—N7	93.36 (9)	N7—C33—C22	119.1 (3)
N3—Co1—N5	93.28 (9)	N7—C33—C34	120.3 (3)
N3—Co1—N6	92.63 (9)	C22—C33—C34	120.6 (3)
N3—Co1—N7	178.92 (9)	C33—C34—C35	120.0 (3)
N5—Co1—N6	88.96 (8)	C34—C35—C36	119.1 (3)
N5—Co1—N7	85.91 (9)	C35—C36—C37	118.5 (3)
N6—Co1—N7	86.65 (9)	N7—C37—C36	122.8 (3)
N9—Co2—N10	111.04 (11)	N8—C38—C22	117.6 (2)
N9—Co2—N11	110.63 (11)	N8—C38—C39	121.5 (3)
N9—Co2—N12	107.81 (11)	C22—C38—C39	120.3 (3)
N10—Co2—N11	109.15 (11)	C38—C39—C40	119.6 (3)
N10—Co2—N12	111.24 (10)	C39—C40—C41	119.2 (3)
N11—Co2—N12	106.88 (11)	C40—C41—C42	117.6 (3)
O3—O1—C47	60.6 (13)	N8—C42—C41	124.5 (3)
O1—O3—C47	71.1 (14)	S1—C43—N9	178.6 (3)
Co1—N1—C2	118.89 (14)	S2—C44—N10	179.1 (3)
Co1—N1—C6	119.8 (2)	S3—C45—N11	178.9 (3)
C2—N1—C6	118.8 (3)	S4—C46—N12	178.7 (3)
Co1—N2—C7	120.28 (17)	O1—C47—O2	122.7 (11)
Co1—N2—C11	119.75 (19)	O1—C47—O3	48.3 (9)
C7—N2—C11	118.6 (3)	O2—C47—O3	96.8 (13)
Co1—N3—C12	120.76 (17)	C2—C3—H1	120.648
Co1—N3—C16	119.70 (19)	C4—C3—H1	120.639
C12—N3—C16	119.4 (3)	C3—C4—H2	120.093
C17—N4—C21	117.6 (3)	C5—C4—H2	120.088
Co1—N5—C23	119.30 (14)	C4—C5—H3	120.835
Co1—N5—C27	119.56 (19)	C6—C5—H3	120.840
C23—N5—C27	119.4 (2)	N1—C6—H4	118.707
Co1—N6—C28	120.00 (17)	C5—C6—H4	118.717
Co1—N6—C32	119.59 (19)	C7—C8—H5	120.059
C28—N6—C32	118.9 (2)	C9—C8—H5	120.060
Co1—N7—C33	120.10 (17)	C8—C9—H6	120.288
Co1—N7—C37	120.5 (2)	C10—C9—H6	120.272
C33—N7—C37	119.2 (3)	C9—C10—H7	121.141
C38—N8—C42	117.5 (3)	C11—C10—H7	121.113
Co2—N9—C43	169.4 (3)	N2—C11—H8	118.509
Co2—N10—C44	171.0 (3)	C10—C11—H8	118.521
Co2—N11—C45	174.2 (3)	C12—C13—H9	119.818
Co2—N12—C46	174.0 (3)	C14—C13—H9	119.815
C2—C1—C7	102.5 (2)	C13—C14—H10	120.331
C2—C1—C12	110.85 (18)	C15—C14—H10	120.338
C2—C1—C17	114.5 (2)	C14—C15—H11	121.052
C7—C1—C12	112.1 (2)	C16—C15—H11	121.040
C7—C1—C17	111.4 (2)	N3—C16—H12	118.388
C12—C1—C17	105.6 (2)	C15—C16—H12	118.404
N1—C2—C1	114.6 (2)	C17—C18—H13	121.038
N1—C2—C3	121.6 (2)	C19—C18—H13	121.048
C1—C2—C3	123.2 (3)	C18—C19—H14	119.260
C2—C3—C4	118.7 (3)	C20—C19—H14	119.262
C3—C4—C5	119.8 (3)	C19—C20—H15	121.247
C4—C5—C6	118.3 (3)	C21—C20—H15	121.246
N1—C6—C5	122.6 (3)	N4—C21—H16	118.606
N2—C7—C1	116.6 (3)	C20—C21—H16	118.611
N2—C7—C8	120.8 (3)	C23—C24—H17	120.344
C1—C7—C8	122.2 (3)	C25—C24—H17	120.348
C7—C8—C9	119.9 (3)	C24—C25—H18	120.300
C8—C9—C10	119.4 (3)	C26—C25—H18	120.294
C9—C10—C11	117.7 (3)	C25—C26—H19	120.921
N2—C11—C10	123.0 (3)	C27—C26—H19	120.902
N3—C12—C1	118.7 (3)	N5—C27—H20	118.623
N3—C12—C13	119.7 (3)	C26—C27—H20	118.626
C1—C12—C13	121.6 (3)	C28—C29—H21	120.376
C12—C13—C14	120.4 (3)	C30—C29—H21	120.354
C13—C14—C15	119.3 (3)	C29—C30—H22	120.399
C14—C15—C16	117.9 (3)	C31—C30—H22	120.404
N3—C16—C15	123.2 (3)	C30—C31—H23	120.602
N4—C17—C1	118.2 (2)	C32—C31—H23	120.610
N4—C17—C18	122.6 (3)	N6—C32—H24	118.943
C1—C17—C18	118.5 (3)	C31—C32—H24	118.949
C17—C18—C19	117.9 (3)	C33—C34—H25	120.015
C18—C19—C20	121.5 (3)	C35—C34—H25	120.000
C19—C20—C21	117.5 (3)	C34—C35—H26	120.458
N4—C21—C20	122.8 (3)	C36—C35—H26	120.461
C23—C22—C28	103.8 (2)	C35—C36—H27	120.743
C23—C22—C33	109.86 (19)	C37—C36—H27	120.750
C23—C22—C38	115.9 (2)	N7—C37—H28	118.600
C28—C22—C33	111.8 (2)	C36—C37—H28	118.616
C28—C22—C38	110.2 (2)	C38—C39—H29	120.186
C33—C22—C38	105.4 (2)	C40—C39—H29	120.183
N5—C23—C22	113.9 (2)	C39—C40—H30	120.402
N5—C23—C24	120.9 (3)	C41—C40—H30	120.408
C22—C23—C24	124.9 (3)	C40—C41—H31	121.178
C23—C24—C25	119.3 (3)	C42—C41—H31	121.180
C24—C25—C26	119.4 (3)	N8—C42—H32	117.763
C25—C26—C27	118.2 (3)	C41—C42—H32	117.774
			
N1—Co1—N2—C7	−32.62 (14)	C38—N8—C42—C41	1.0 (5)
N1—Co1—N2—C11	133.93 (14)	C42—N8—C38—C22	172.2 (3)
N2—Co1—N1—C2	33.86 (16)	C42—N8—C38—C39	1.0 (4)
N2—Co1—N1—C6	−128.18 (16)	C2—C1—C7—N2	75.6 (3)
N1—Co1—N3—C12	41.63 (13)	C2—C1—C7—C8	−97.0 (3)
N1—Co1—N3—C16	−134.87 (13)	C7—C1—C2—N1	−72.0 (3)
N3—Co1—N1—C2	−53.64 (16)	C7—C1—C2—C3	99.2 (3)
N3—Co1—N1—C6	144.32 (16)	C2—C1—C12—N3	−63.2 (3)
N1—Co1—N6—C28	−150.37 (14)	C2—C1—C12—C13	115.6 (3)
N1—Co1—N6—C32	43.69 (14)	C12—C1—C2—N1	47.8 (3)
N6—Co1—N1—C2	−146.15 (16)	C12—C1—C2—C3	−141.1 (3)
N6—Co1—N1—C6	51.82 (16)	C2—C1—C17—N4	−33.7 (3)
N1—Co1—N7—C33	140.95 (14)	C2—C1—C17—C18	156.07 (19)
N1—Co1—N7—C37	−43.96 (14)	C17—C1—C2—N1	167.16 (19)
N7—Co1—N1—C2	127.06 (16)	C17—C1—C2—C3	−21.7 (4)
N7—Co1—N1—C6	−34.97 (16)	C7—C1—C12—N3	50.7 (3)
N2—Co1—N3—C12	−46.63 (13)	C7—C1—C12—C13	−130.5 (2)
N2—Co1—N3—C16	136.87 (13)	C12—C1—C7—N2	−43.3 (3)
N3—Co1—N2—C7	53.23 (14)	C12—C1—C7—C8	144.12 (19)
N3—Co1—N2—C11	−140.23 (14)	C7—C1—C17—N4	−149.48 (18)
N2—Co1—N5—C23	146.89 (16)	C7—C1—C17—C18	40.2 (3)
N2—Co1—N5—C27	−48.33 (16)	C17—C1—C7—N2	−161.38 (17)
N5—Co1—N2—C7	146.45 (14)	C17—C1—C7—C8	26.0 (3)
N5—Co1—N2—C11	−47.00 (14)	C12—C1—C17—N4	88.6 (2)
N2—Co1—N7—C33	−130.68 (14)	C12—C1—C17—C18	−81.7 (3)
N2—Co1—N7—C37	44.40 (14)	C17—C1—C12—N3	172.23 (16)
N7—Co1—N2—C7	−127.58 (14)	C17—C1—C12—C13	−9.0 (3)
N7—Co1—N2—C11	38.97 (14)	N1—C2—C3—C4	−3.5 (4)
N3—Co1—N5—C23	−125.68 (16)	C1—C2—C3—C4	−174.1 (2)
N3—Co1—N5—C27	39.10 (16)	C2—C3—C4—C5	0.8 (4)
N5—Co1—N3—C12	−137.68 (13)	C3—C4—C5—C6	0.8 (4)
N5—Co1—N3—C16	45.82 (13)	C4—C5—C6—N1	0.1 (5)
N3—Co1—N6—C28	123.79 (14)	N2—C7—C8—C9	7.0 (3)
N3—Co1—N6—C32	−42.14 (14)	C1—C7—C8—C9	179.27 (17)
N6—Co1—N3—C12	133.22 (13)	C7—C8—C9—C10	−0.3 (4)
N6—Co1—N3—C16	−43.29 (13)	C8—C9—C10—C11	−4.3 (4)
N5—Co1—N6—C28	30.56 (14)	C9—C10—C11—N2	2.7 (4)
N5—Co1—N6—C32	−135.37 (14)	N3—C12—C13—C14	−1.1 (3)
N6—Co1—N5—C23	−33.11 (16)	C1—C12—C13—C14	−179.84 (17)
N6—Co1—N5—C27	131.68 (16)	C12—C13—C14—C15	0.1 (4)
N5—Co1—N7—C33	−39.72 (14)	C13—C14—C15—C16	0.0 (4)
N5—Co1—N7—C37	135.36 (14)	C14—C15—C16—N3	0.9 (4)
N7—Co1—N5—C23	53.61 (16)	N4—C17—C18—C19	5.0 (4)
N7—Co1—N5—C27	−141.61 (16)	C1—C17—C18—C19	174.80 (19)
N6—Co1—N7—C33	49.47 (14)	C17—C18—C19—C20	−2.6 (4)
N6—Co1—N7—C37	−135.45 (14)	C18—C19—C20—C21	−0.1 (4)
N7—Co1—N6—C28	−55.41 (14)	C19—C20—C21—N4	0.6 (5)
N7—Co1—N6—C32	138.66 (14)	C23—C22—C28—N6	−76.4 (3)
O3—O1—C47—O2	−67.4 (13)	C23—C22—C28—C29	98.4 (3)
O3—O1—C47—O3	0.0 (13)	C28—C22—C23—N5	71.0 (3)
C47—O1—O3—C47	0.00 (19)	C28—C22—C23—C24	−102.2 (3)
O1—O3—C47—O1	0.00 (15)	C23—C22—C33—N7	66.2 (3)
O1—O3—C47—O2	128.5 (9)	C23—C22—C33—C34	−112.5 (3)
Co1—N1—C2—C1	13.5 (3)	C33—C22—C23—N5	−48.7 (3)
Co1—N1—C2—C3	−157.77 (15)	C33—C22—C23—C24	138.1 (3)
Co1—N1—C6—C5	159.32 (18)	C23—C22—C38—N8	29.4 (3)
C2—N1—C6—C5	−2.7 (4)	C23—C22—C38—C39	−159.3 (2)
C6—N1—C2—C1	175.7 (2)	C38—C22—C23—N5	−168.0 (2)
C6—N1—C2—C3	4.4 (4)	C38—C22—C23—C24	18.8 (4)
Co1—N2—C7—C1	−14.6 (3)	C28—C22—C33—N7	−48.5 (3)
Co1—N2—C7—C8	158.10 (13)	C28—C22—C33—C34	132.8 (2)
Co1—N2—C11—C10	−163.01 (14)	C33—C22—C28—N6	41.9 (3)
C7—N2—C11—C10	3.8 (3)	C33—C22—C28—C29	−143.27 (19)
C11—N2—C7—C1	178.71 (17)	C28—C22—C38—N8	146.82 (19)
C11—N2—C7—C8	−8.6 (3)	C28—C22—C38—C39	−41.9 (3)
Co1—N3—C12—C1	4.3 (3)	C38—C22—C28—N6	158.87 (17)
Co1—N3—C12—C13	−174.53 (12)	C38—C22—C28—C29	−26.3 (3)
Co1—N3—C16—C15	174.58 (14)	C33—C22—C38—N8	−92.4 (3)
C12—N3—C16—C15	−2.0 (3)	C33—C22—C38—C39	78.9 (3)
C16—N3—C12—C1	−179.21 (17)	C38—C22—C33—N7	−168.27 (17)
C16—N3—C12—C13	2.0 (3)	C38—C22—C33—C34	13.1 (3)
C17—N4—C21—C20	1.6 (4)	N5—C23—C24—C25	3.7 (4)
C21—N4—C17—C1	−174.3 (2)	C22—C23—C24—C25	176.5 (3)
C21—N4—C17—C18	−4.5 (4)	C23—C24—C25—C26	−0.8 (4)
Co1—N5—C23—C22	−13.1 (3)	C24—C25—C26—C27	−1.2 (4)
Co1—N5—C23—C24	160.44 (15)	C25—C26—C27—N5	0.6 (5)
Co1—N5—C27—C26	−162.57 (17)	N6—C28—C29—C30	−5.3 (4)
C23—N5—C27—C26	2.2 (4)	C22—C28—C29—C30	−179.88 (18)
C27—N5—C23—C22	−177.9 (2)	C28—C29—C30—C31	−1.3 (4)
C27—N5—C23—C24	−4.4 (4)	C29—C30—C31—C32	5.6 (4)
Co1—N6—C28—C22	16.1 (3)	C30—C31—C32—N6	−3.7 (4)
Co1—N6—C28—C29	−158.71 (13)	N7—C33—C34—C35	2.5 (3)
Co1—N6—C32—C31	163.28 (14)	C22—C33—C34—C35	−178.80 (17)
C28—N6—C32—C31	−2.8 (3)	C33—C34—C35—C36	0.6 (4)
C32—N6—C28—C22	−177.87 (17)	C34—C35—C36—C37	−2.3 (4)
C32—N6—C28—C29	7.3 (3)	C35—C36—C37—N7	1.0 (4)
Co1—N7—C33—C22	−7.4 (3)	N8—C38—C39—C40	−2.3 (4)
Co1—N7—C33—C34	171.31 (12)	C22—C38—C39—C40	−173.3 (2)
Co1—N7—C37—C36	−173.03 (14)	C38—C39—C40—C41	1.7 (4)
C33—N7—C37—C36	2.1 (4)	C39—C40—C41—C42	0.2 (4)
C37—N7—C33—C22	177.50 (17)	C40—C41—C42—N8	−1.6 (5)
C37—N7—C33—C34	−3.8 (3)		

Symmetry codes: (i) *x*, *y*, *z*+1; (ii) −*x*+1, *y*−1/2, −*z*; (iii) *x*, *y*, *z*−1; (iv) *x*−1, *y*, *z*; (v) −*x*+1, *y*+1/2, −*z*; (vi) *x*−1, *y*, *z*−1; (vii) −*x*+1, *y*−1/2, −*z*+1; (viii) *x*+1, *y*, *z*+1; (ix) *x*+1, *y*, *z*; (x) −*x*+2, *y*+1/2, −*z*+1; (xi) −*x*+1, *y*+1/2, −*z*+1; (xii) −*x*+2, *y*−1/2, −*z*+1; (xiii) *x*−1, *y*, *z*+1; (xiv) −*x*+2, *y*−1/2, −*z*; (xv) −*x*+2, *y*+1/2, −*z*; (xvi) *x*+1, *y*, *z*−1.
